# Enhancing excitatory activity of somatosensory cortex alleviates neuropathic pain through regulating homeostatic plasticity

**DOI:** 10.1038/s41598-017-12972-6

**Published:** 2017-10-06

**Authors:** Wenhui Xiong, Xingjie Ping, Matthew S. Ripsch, Grace Santa Cruz Chavez, Heidi Elise Hannon, Kewen Jiang, Chunhui Bao, Vaishnavi Jadhav, Lifang Chen, Zhi Chai, Cungen Ma, Huangan Wu, Jianqiao Feng, Armin Blesch, Fletcher A. White, Xiaoming Jin

**Affiliations:** 1 Department of Anatomy and Cell Biology, Indiana University School of Medicine, Indianapolis, IN 46202 USA; 2 Department of Anesthesia, Indiana University School of Medicine, Indianapolis, IN 46202 USA; 3 Department of Neurological Surgery, Indiana University School of Medicine, Indianapolis, IN 46202 USA; 4 Spinal Cord and Brain Injury Research Group, Stark Neurosciences Research Institute. Indiana University School of Medicine, Indianapolis, IN 46202 USA; 5 Department of Biomedical Engineering, Purdue School of Engineering and Technology. IUPUI, Indianapolis, USA; 6 Department of Neurology, Children’s Hospital, Zhejiang University School of Medicine, Hangzhou, China; 7 Shanghai Research Institute of Acupuncture-Moxibustion and Meridian, Shanghai, China; 8 Department of Acupuncture, Zhejiang Traditional Chinese Medical University and the Third Affiliated Hospital, Hangzhou, Zhejiang, China; 9 Research Center of Neurobiology, Shanxi University of Traditional Chinese Medicine, Taiyuan, China; 10 Research and Development Services, Richard L. Roudebush VA Medical Center, Indianapolis, IN 46202 USA

## Abstract

Central sensitization and network hyperexcitability of the nociceptive system is a basic mechanism of neuropathic pain. We hypothesize that development of cortical hyperexcitability underlying neuropathic pain may involve homeostatic plasticity in response to lesion-induced somatosensory deprivation and activity loss, and can be controlled by enhancing cortical activity. In a mouse model of neuropathic pain, *in vivo* two-photon imaging and patch clamp recording showed initial loss and subsequent recovery and enhancement of spontaneous firings of somatosensory cortical pyramidal neurons. Unilateral optogenetic stimulation of cortical pyramidal neurons both prevented and reduced pain-like behavior as detected by bilateral mechanical hypersensitivity of hindlimbs, but corpus callosotomy eliminated the analgesic effect that was ipsilateral, but not contralateral, to optogenetic stimulation, suggesting involvement of inter-hemispheric excitatory drive in this effect. Enhancing activity by focally blocking cortical GABAergic inhibition had a similar relieving effect on the pain-like behavior. Patch clamp recordings from layer V pyramidal neurons showed that optogenetic stimulation normalized cortical hyperexcitability through changing neuronal membrane properties and reducing frequency of excitatory postsynaptic events. We conclude that development of neuropathic pain involves abnormal homeostatic activity regulation of somatosensory cortex, and that enhancing cortical excitatory activity may be a novel strategy for preventing and controlling neuropathic pain.

## Introduction

Neuropathic pain is a major public health problem that affects 7–10% of the general population^1^. It is often caused by a primary lesion of the nervous system such as nerve or spinal cord injury, which leads to maladaptive plasticity and central sensitization of the nociceptive pathways^2^. The resulting neuronal hyperexcitability and ectopic spontaneous firing are believed to be key pathophysiological mechanisms^2,3^. Accordingly, suppressing such hyperexcitability and aberrant activity by directly inhibiting network excitability or enhancing inhibition is a generally accepted paradigm for the management of neuropathic pain^4^. However, the current pharmacological treatments only produce partial pain relief in a portion of the patients^5^.

Injury-induced loss of cortical activity is known to trigger homeostatic regulation of activity, a well-established mechanism in which cortical neurons dynamically regulate their synaptic strengths and intrinsic properties in response to an imposed increase or decrease of synaptic input so that a relatively constant activity level is maintained^6–10^

Thus, hyperexcitability, which underlies neuropathic pain, may be initiated and maintained via a homeostatic mechanism that more than compensates for loss of input from injured pathways. After some types of spinal cord injury, primary somatosensory cortex (S1) exhibits initial activity loss and subsequent hyperexcitability and paroxysmal discharges^11–13^, consistent with homeostatic activity regulation. Because the injury-induced changes are often permanent or progressive, such homeostatic compensation likely results from constant or progressive loss of activity in the associated cortex. In turn, restoration of this activity by cortical stimulation would reduce pathological homeostatic regulation and control neuropathic pain. Indeed, cortical stimulation techniques such as motor cortical stimulation and repetitive transcranial magnetic stimulation have been used for patients with refractory neuropathic pain^14,15^. Although studies suggest that activation of brain inhibitory pathways or descending projections may contribute to the analgesic effect, the mechanisms are poorly understood^16,17^. Particularly, the direct effect of cortical stimulation on the neurophysiology of cortical neurons themselves has not been directly investigated.

Using a transient spinal cord ischemia model (tSCI) of neuropathic pain in mice^18,19^, we studied the manifestation of homeostatic plasticity in the S1 *in vitro* and *in vivo*, and demonstrated that optogenetic or pharmacological enhancement of cortical activity diminished injury-induced behavioral hypersensitivity in this model. Mechanistically, we used patch clamp recording to demonstrate changes in neuronal intrinsic properties and synaptic transmission after injury, and the effect of optogenetic stimulation in reversing most of the injury-induced electrophysiological alterations. Our results suggest that the development of neuropathic pain involves homeostatic regulation and that enhancing cortical excitatory activity can control neuropathic pain.

## Results

### Homeostatic regulation of spontaneous activity after tSCI *in vivo* and *in vitro*

**Figure 1:**
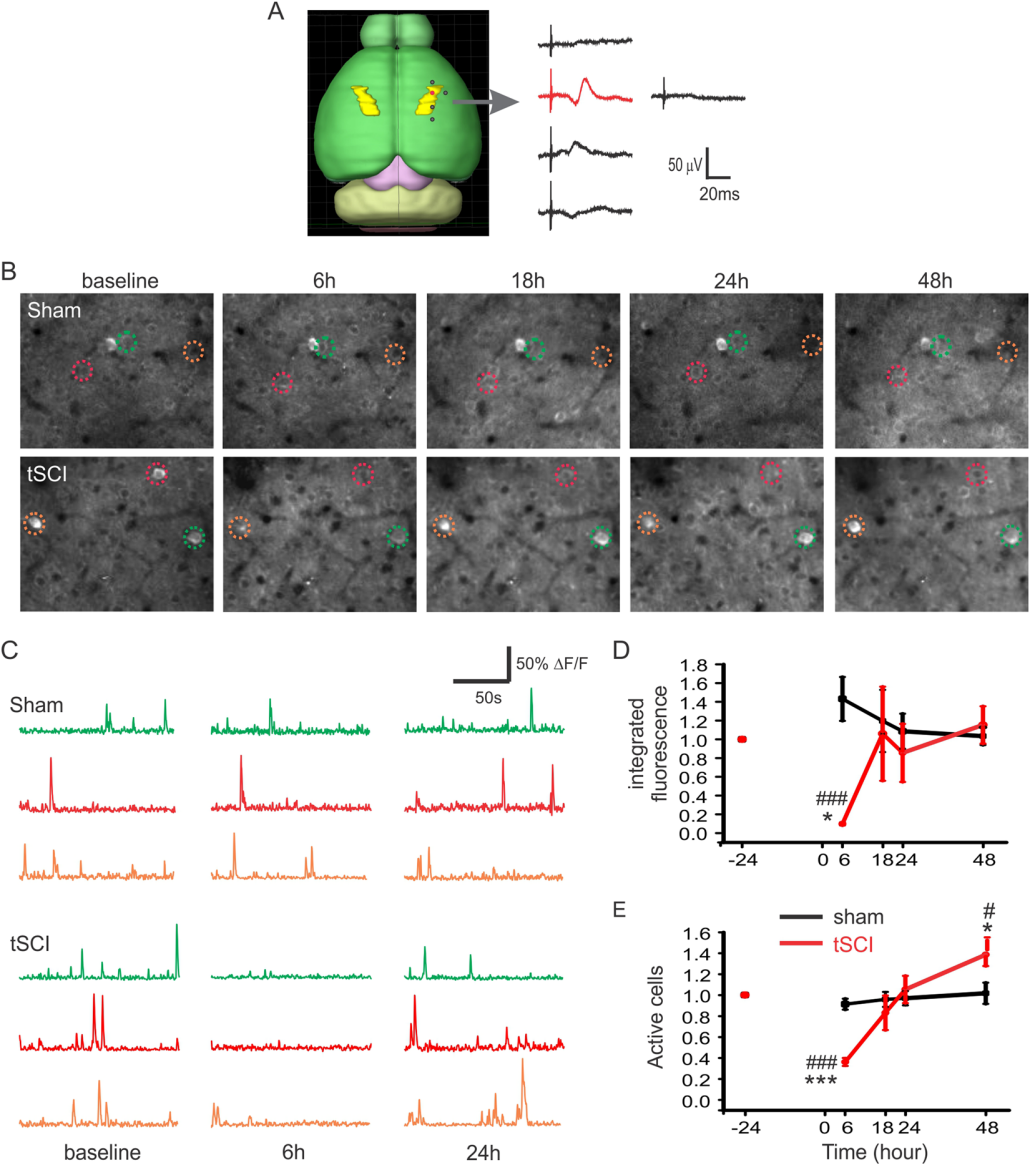
Repeated *in vivo* two-photon imaging revealed S1 activity changes of adult mice after tSCI. (**A)** The location of hindlimb S1 area (HL-S1, yellow regions) was identified by applying electrical stimulation (0.2 mA and 200 μs) to the hind paw and recording cortical sensory evoked potentials *in vivo* (n = 4 mice). Each dot on the right cortical surface corresponds to a recording trace on the right. The distances between neighboring traces were 1 mm. A rectangular HL-S1 area was found to span 1.5–2 mm laterally and 2.5–3 mm in an anterolateral and posteromedial direction, with the medial border being ~1 mm lateral to the midline and the longitudinal center being ~1 mm posterior to the bregma. The mouse brain image was created with Allen Mouse Brain Atlas using Brain Explorer® 2 (©2014 Allen Institute for Brain Science. Allen Mouse Brain Atlas: http://mouse.brain-map.org/). (**B**) Average projections of the same regions of cortical layer II/III GCaMP6-expressing neurons at different time points after sham (top) and tSCI (bottom) surgery. (**C**) ΔF/F traces of calcium transients of neurons of sham and tSCI groups that correspond to the color-circled neurons in (**A**). (**D)** Changes in mean integrated fluorescence of the tSCI and sham groups indicate a loss of neuronal activity at 6 hours post-tSCI followed by recovery, suggesting homeostatic regulation of activity after tSCI *in vivo* (n = 7 mice in each group). (**E**) There is a similar pattern of change when the ratios of active neurons are quantified and compared. However, the ratio of active neurons in tSCI group at 48 hours was significantly higher than that of the sham group and the baseline of the tSCI group. For graphs (**D** and **E**), repeated measure ANOVA was followed by Bonferroni test. *p < 0.05 and ***p < 0.001 when compared with the baseline of the tSCI group; #p < 0.05 and ###p < 0.001 when compared between sham and tSCI groups at corresponding time points.

We first identified the location of the hindlimb area of the S1 (HL-S1) based on the maximum amplitude of sensory-evoked potential (SEP) induced by electrically stimulating the hind paw of naïve mice *in vivo* (n = 4 mice). A rectangular HL-S1 area was found to span 1.5–2 mm laterally and 2 mm in an anterolateral and posteromedial direction, with the medial border being ~1 mm lateral to the midline and the longitudinal center being ~1 mm posterior to the bregma (Fig. 1A). All subsequent *in vitro* and *in vivo* experiments in this study were targeted to this cortical region.

To determine whether and how a lesion to the somatosensory pathway affects neuronal activity of S1 *in vivo*, we used a repeated two-photon imaging technique to measure activity changes of cortical layer II/III pyramidal neurons in GCaMP6s transgenic mice that were sedated with chlorprothixene^20,21^. The HL-S1 area of each mouse was repeatedly imaged at baseline and 6, 18, 24, and 48 hours after sham injury or tSCI (Fig. 1B-C, 356 cells from 7 sham-injured mice and 327 cells from 7 tSCI mice). Under baseline or sham-injured conditions, activities of fluorescent cells were clearly visible. At 6 hours post-tSCI, both the mean integrated fluorescence (for measuring intensity of spiking activity of individual neurons) and fraction of active neurons (those that fired at least once during the imaging period) were dramatically reduced when compared with the sham group or baseline level of the tSCI group (Fig. 1B–E, repeated-measurement ANOVA followed by Bonferroni tests, *p* < 0.05-0.001, n = 7 mice in each group), suggesting an initial loss of activity. They recovered to about baseline levels in 18 and 24 hours after injury, but the ratio of active neurons in the tSCI group became significantly higher than its baseline level and that of the sham group at 48 hours (both *p* < 0.05). In contrast, there were no significant changes at different time points in integrated fluorescence and ratio of active cells in the sham group.

**Figure 2:**
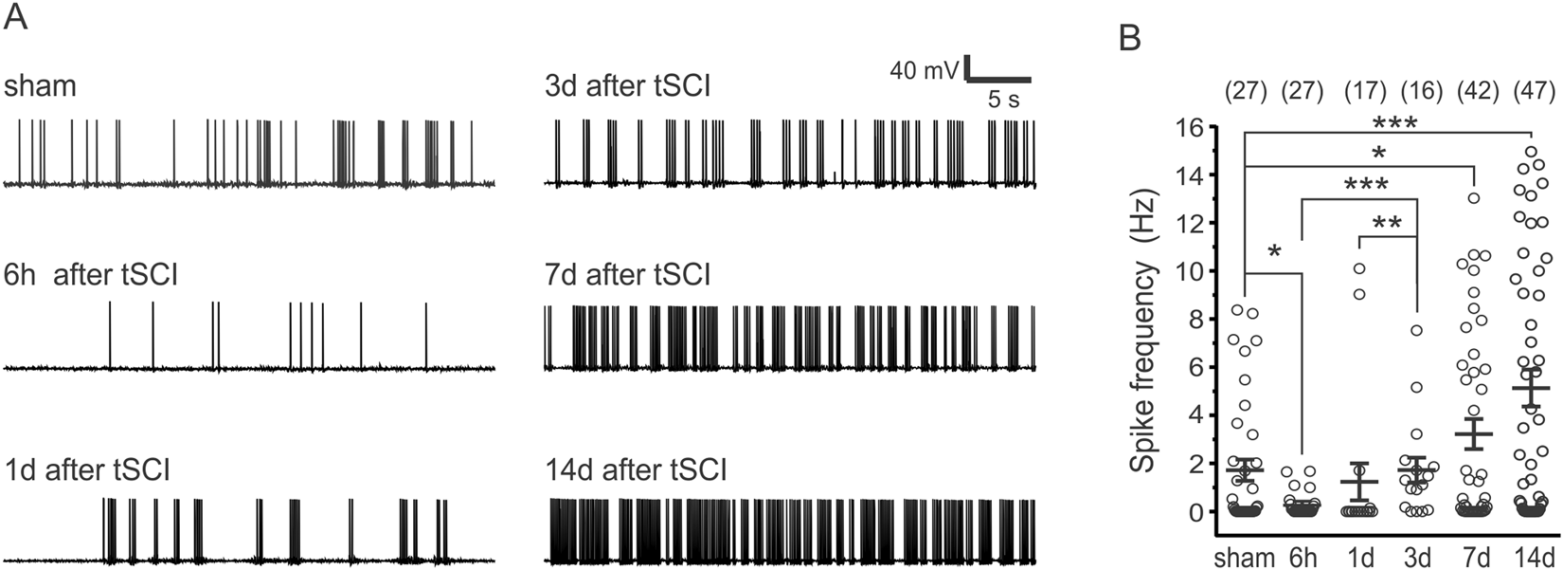
Development of elevated spontaneous activity in S1 of the tSCI mice. (**A**).Representative traces of spontaneous AP firing in layer V pyramidal neurons in cortical slices from the HL-S1 of the sham group and at different times post-tSCI. Recordings were made at −60 mV in a modified ACSF that slightly enhanced spontaneous firing (n = 4–8 mice in each group). (**B**) Spike frequencies in different groups (1.72 ± 0.44 Hz, 0.26 ± 0.10 Hz, 1.23 ± 0.77 Hz, 1.72 ± 0.52 Hz, 3.22 ± 0.63 Hz, and 5.13 ± 0.77 Hz for the sham, 6 hour, 1 day, 3 day, 7 day and 14 day groups respectively). There was a significant decrease in spike frequency at 6 hours post-tSCI (*p* < 0.05 when compared with the sham group), which was followed by a progressive increase in spike frequency between 3–14 days post-tSCI. Spike frequency in the 3 day group was higher than 6 hour and 1 day groups, and spike frequencies in 7 day and 14 day groups were higher than the sham, 6 hour, and 1 day groups. *p < 0.05; **p < 0.01; *** < 0.001; Mann-Whitney test). The numbers on top are numbers of recorded neurons in each group.

We further recorded spontaneous action potential (AP) firing of layer V pyramidal neurons in brain slices from the HL-S1 in a modified artificial cerebrospinal fluid (ACSF) that slightly increases neuronal activity^22,23^. The spike frequency at 6 hours after tSCI became significantly lower (Fig. 2A,B, 1.72 ± 0.44 and 0.26 ± 0.10 Hz for sham and 6-hour groups respectively, *p* < 0.05, Mann-Whitney test, n = 4–8 mice in each group), and recovered at 1 day and 3 days post-tSCI (*p* < 0.001 and *p* < 0.01 respectively, Mann-Whitney test). At 7 days and 14 days post-tSCI, the frequencies were significantly higher than the sham and 6 hours post-tSCI groups (3.22 ± 0.63 and 5.13 ± 0.77 Hz, *p* < 0.05 and *p* < 0.001 respectively, Mann-Whitney test). Thus, the observed *in vivo* and *in vitro* transitions from initial activity loss to enhanced spontaneous activity suggest a homeostatic regulation of cortical activity following tSCI.

### Optogenetic stimulation of the S1 prevented pain-like behavior in two models of neuropathic pain

**Figure 3:**
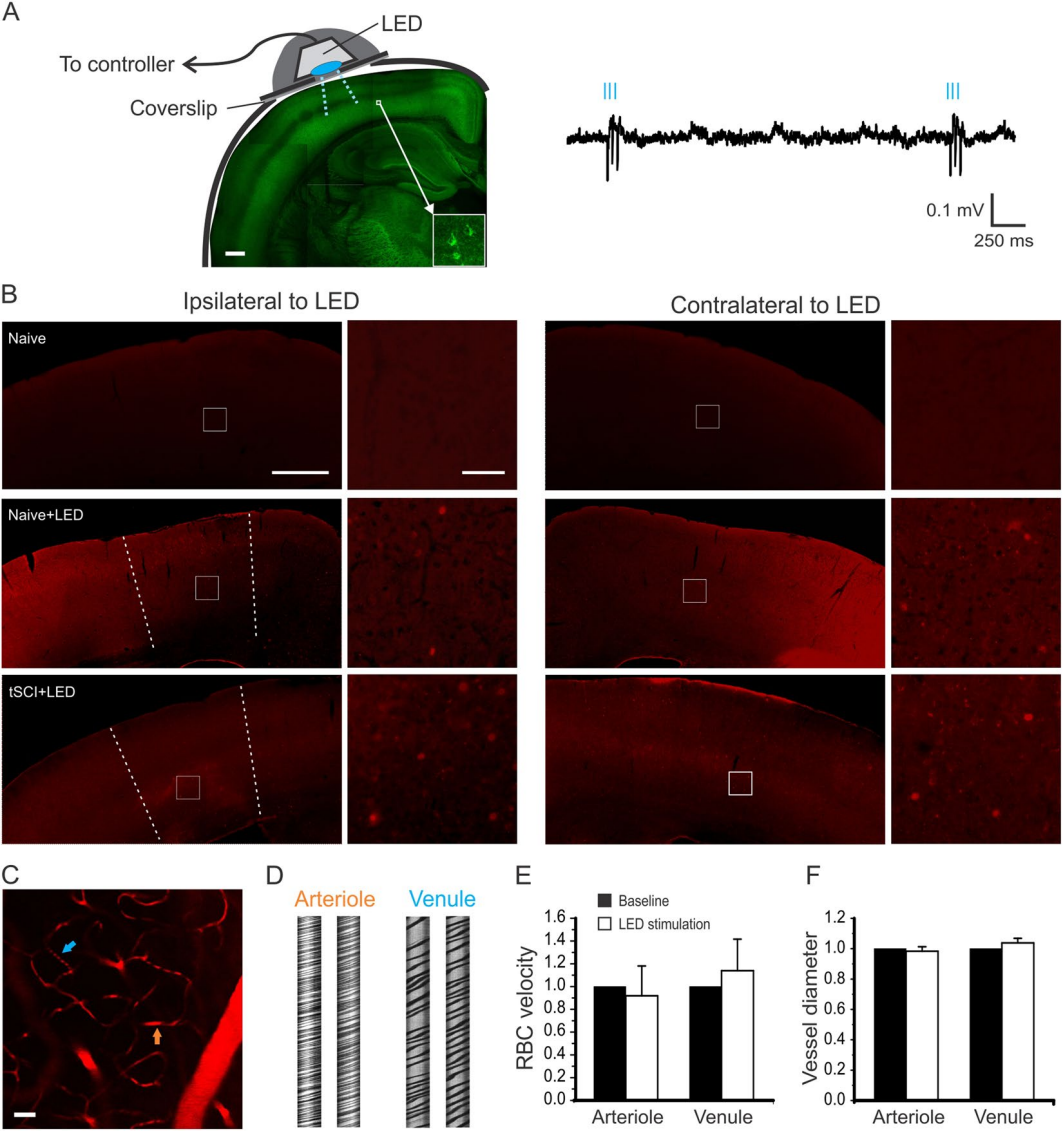
Activation of S1 neurons using optogenetic stimulation in ChR2 mice. (**A**) Chronic optogenetic stimulation was made by implanting a blue LED (475 nm) on a cranial window over the S1 area of the Thy1-ChR2-YFP transgenic mice (left). About 1.5 mm diameter area of the window was exposed to the LED, with the surrounding area being blocked by opaque tape. Cortical expression of ChR2 is mostly in layer V pyramidal neurons (insert). The effectiveness of LED stimulation was confirmed by the evoked spikes of EEG recording (right) *in vivo*. (**B**) C-fos staining of cortical slices indicates activation of cortical neurons by the optogenetic stimulation. There was little staining in the cortex of naïve mice without optogenetic stimulation (top panel). For each row, c-fos staining of the ipsilateral (left two columns) and contralateral (right two columns) cortex are shown; enlarged views of the white square areas are shown to the right. The dotted lines indicate approximate borders of activated cortical neurons. After turning on blue light pulses (see methods) for 1.5 hours, there were c-fos positive cells in layer II/III and V in the S1 regions of both ipsilateral and contralateral hemispheres in uninjured ChR2 mice (middle panel). Similar bilateral c-fos staining pattern was seen in tSCI mice that received 1.5 hours LED stimulation (bottom panel). **C**–**F** No significant changes in cerebral vessel diameter and blood flow after light stimulation. Cerebral vessels were visualized by intravenous injection of sulforhodamine 101 in wild C57BL mice and imaged with two-photon microscopy (**C**). Cortical superficial arterioles and venules were identified and imaged in line scan mode (**D**). After 15 minutes of blue LED light stimulation, there were no significant changes in the velocities of red blood cells (RBC) and vessel diameters of arterioles and venules (**E** and **F**, n = 4 mice). Scale bars: A = 400 µm; B (1^st^ and 3^rd^ columns) = 500 µm; B (2^nd^ and 4^th^ columns) = 50 µm; C = 80 µm.

To specifically activate cortical excitatory neurons, we used a miniature blue light-emitting diode (LED, ~470 nm peak wavelength) to optogenetically stimulate S1 activity in ChR2 transgenic mice in which the cortical expression of ChR2 is mostly in layer V pyramidal neurons (Fig. 3A).

The LED was driven by a wired miniature circuit we made to generate a burst of 3 pulses at 33 Hz every 3 seconds. To confirm the effectiveness of LED stimulation, we recorded EEG and found that the light pulses induced a precise bursting response of the cortex (Fig. 3A, n = 3 mice). To further confirm the effect of the light stimulation on activating neurons, we made c-fos staining of cortical slices under different conditions. In naïve, unstimulated mice, few cells were stained by c-fos antibody (Fig. 3B). Following a 1.5 hour bursting stimulation using the LED in either normal uninjured mice or tSCI mice, an increased number of c-fos expressing neurons were visible in cortical layer II/III and layer V of the S1 region, but much less in regions outside of the targeted S1 (Fig. 3B, n = 4 mice in each group), suggesting focal activation of cortical neurons by the optogenetic stimulation.

Because a recent study showed that light stimulation of the brain can induce rapid and transient vasodilation without activating neurons and astrocytes^24^, we tested whether our optogenetic stimulation could change cerebral blood circulation. Cerebral blood vessels were visualized by intravenous injection of a fluorescence dye Sulforhodomine 101 and imaged through a cranial window (Fig. 3C,D). Following 15 minutes of LED stimulation, *in vivo* two-photon imaging of fluorescence labeled cerebral vessels showed no significant changes in the velocity of red blood cells (RBC) and vessel diameters of arterioles and venules between before and after light stimulation (Fig. 3E,F, n = 4 mice in each group). The results suggest that our light stimulation protocol did not induce detectable changes in cerebral blood circulation.

**Figure 4:**
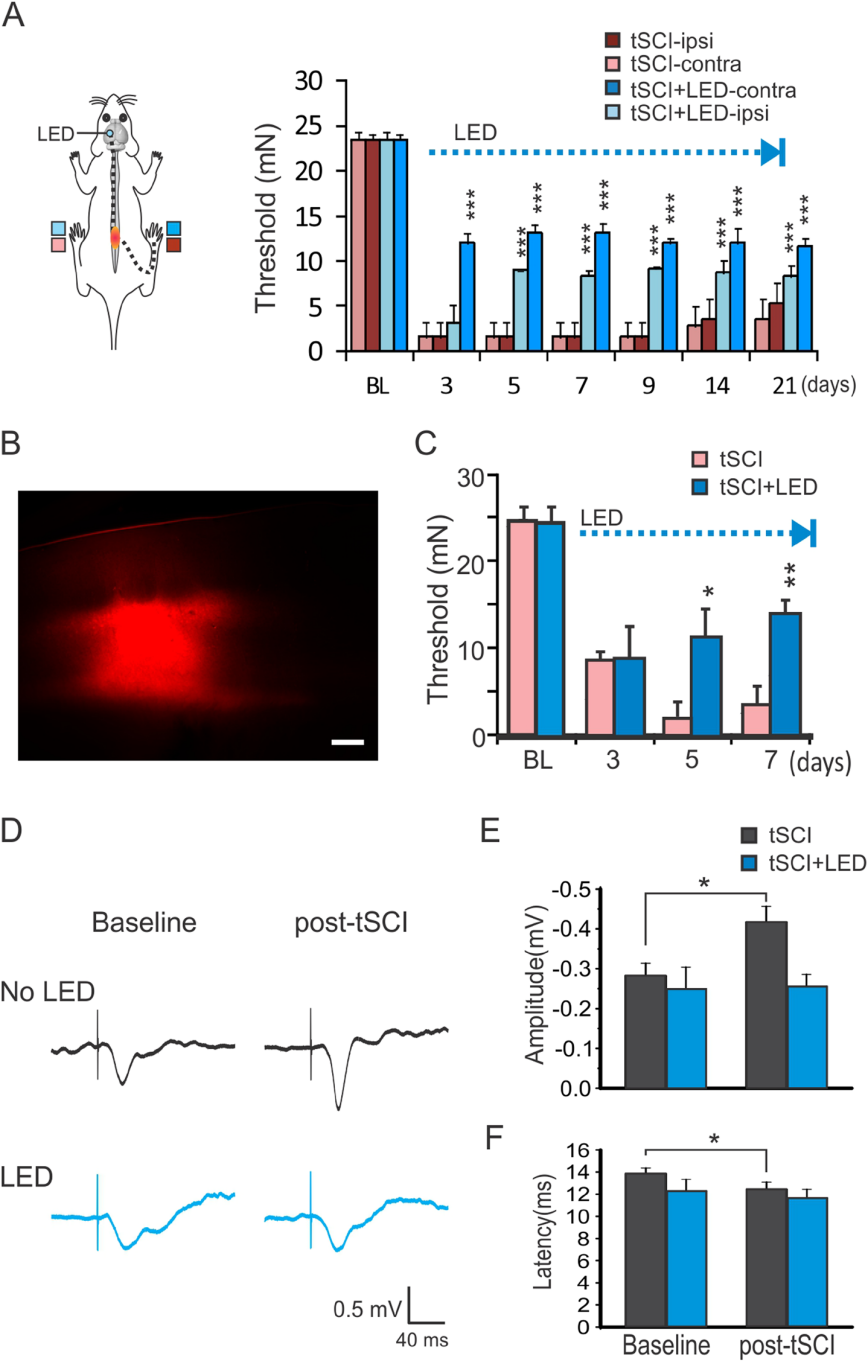
Optogenetic stimulation of S1 prevents the development of mechanical hypersensitivity and reduces cortical hyperexcitability. (**A**) Optogenetic stimulation of the S1 in the tSCI model resulted in significant increases in withdrawal threshold forces of the bilateral hind-paws (***p < 0.001 when stimulated and unstimulated mice were compared; repeated measure ANOVA followed by Bonferroni test, n = 6 mice in each group). (**B**,**C**) Focal S1 optogenetic stimulation in virus transfected mice also induced analgesic effect. Following injection of ChR2-mCherry AAV into the S1 to infect layers II/III and V pyramidal neurons in a focal region (**B**), optogenetic stimulation with the same protocol also induced significant analgesic effect in the tSCI model (**C**). (*p < 0.05; **p < 0.01 when the tSCI + LED group was compared with the tSCI control group; Repeated measure ANOVA, n = 5 mice in each group). Scar bar: 300 µm. (**D**) Sample traces show that tSCI caused a high amplitude of cortical sensory-evoked potential, but optogenetic stimulation with LED for 5 days normalized it. (**E**,**F**) Group data showed that there were higher amplitudes of sensory-evoked response in tSCI group but not tSCI + LED group (**E**) and shorter latency in tSCI group but not tSCI + LED group (**F**). *p < 0.05, paired *t*-test in both E and F (tSCI group: n = 8 mice; tSCI + LED group: n = 5 mice).

The homeostatic plasticity hypothesis predicts that stimulating cortical activity would reduce neuronal hyperexcitability and prevent neuropathic pain. To test this hypothesis, we used the blue LED to optogenetically stimulate S1 of Thy1-ChR2 transgenic mice on the second day after tSCI^25^. Applying the optogenetic stimulation to unilateral S1 for two sessions of 3 hours daily results in a highly significant decrease in mechanical hypersensitivity of bilateral hind-paws, and this effect was significant as early as 3 days after tSCI and remained stable throughout the stimulation period (Fig. 4A, *p* < 0.001 for both sides, repeated-meaure ANOVA followed by Bonferroni tests, n = 6 mice in each group). However, the effect on the ipsilateral hindlimb had a later onset and was smaller in magnitude than the contralateral hindlimb (Fig. 4A).

Optogenetic stimulation of the S1 in ChR2 mice may activate ChR2 axons that pass through the S1 region and activate remote cortical synapses or neuronal somata. To provide more precise stimulation of the S1, we injected a CaMKIIa.hChR2(H134R)-mCherry AAV virus into the S1 and repeated the experiment 2 weeks later. The virus transfected S1 pyramidal neurons within an area of ~1–1.5 mm diameter (Fig. 4B). Optogenetic stimulation with the same protocol induced a significant increase in the threshold of Von Frey test of the contralateral hind paw at day 5 and 7 after tSCI (Fig. 4C, *p* < 0.05 and *p* < 0.01 respectively, repeated-measure ANOVA followed by Bonferroni tests, n = 5 mice in each group). The results support the efficacy of S1 optogenetic stimulation in relieving pain-like behavior in the tSCI model.

To determine the effects of tSCI and optogenetic stimulation on S1 excitability *in vivo*, we recorded cortical sensory evoked potentials (SEPs) before and after tSCI. We found that tSCI caused a significant increase in the amplitude and a significant decrease in the latency of SEPs at 5 days after injury (Fig. 4D–F, *p* < 0.05, paired *t*-test, n = 8 mice). In contrast, optogenetic stimulation for 5 days resulted in unchanged amplitude and latency of the SEPs (Fig. 4D–F, n = 5 mice). The results suggest that optogenetic stimulation of S1 reduces the hyperexcitability of the somatosensory pathway.

**Figure 5:**
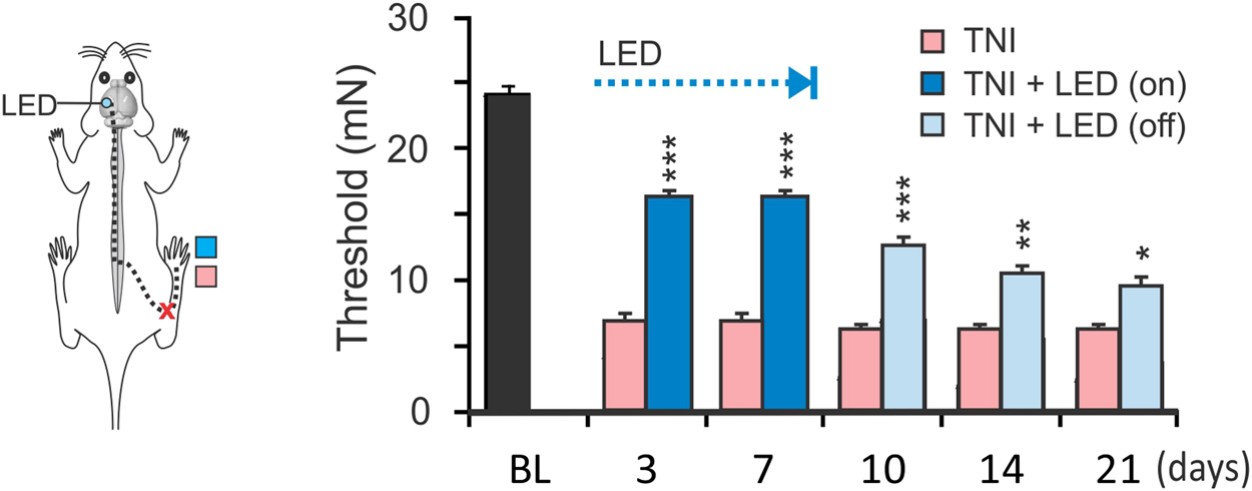
Analgesic effect of optogenetic stimulation in a TNI model: Light stimulation of the S1 for 7 days in a mouse TNI model also resulted in increases in hind-paw withdrawal threshold forces than the sham-injured mice. Furthermore, despite the cessation of the stimulation at day 7, the stimulated mice continued to have a higher pain threshold up to day 21. (*p < 0.05; ***p < 0.001 when compared to the TNI group; Repeated measure ANOVA, n = 6 mice in each group).

We further tested the effect of S1 optogenetic stimulation in a tibial nerve injury (TNI) model of neuropathic pain. Application of the same optogenetic stimulation on S1 for 1 week greatly reduced mechanical hypersensitivity of the contralateral hind-paw, as measured with the Von Frey test (Fig. 5. *p* < 0.001, repeated measure ANOVA followed by Bonferroni test, n = 6 mice in each group). Furthermore, the analgesic effect lasted for > 2 weeks after turning off the LED (Fig. 5. *p* < 0.001 to *p* < 0.05), indicating a lasting effect of the stimulation on controlling hypersensitivity development.

### Optogenetic stimulation of the S1 reduced pain-like behavior via excitatory drive

**Figure 6:**
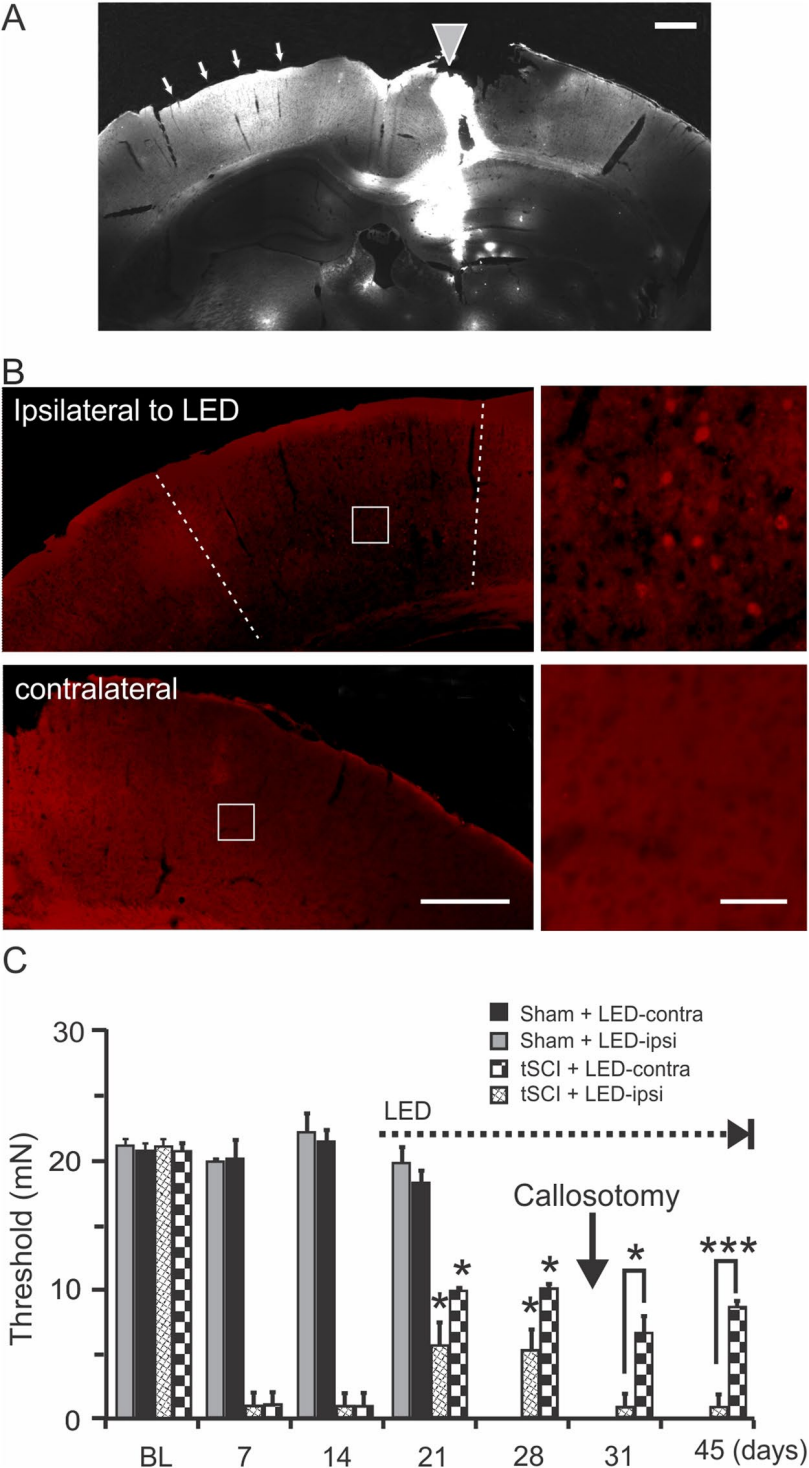
Analgesic effect of S1 optogenetic stimulation is mediated by cortical excitatory drive. (**A**). A corpus callosotomy was made using a “L-shaped” needle coated with a lipophilic fluorescent dye DiI, which labeled both local cut path (gray triangle) and truncated inter-hemispheric axons (arrows) of the intact cortical hemisphere (n = 3 mice). (**B**). In a ChR2 mouse that received callosotomy, c-fos staining shows that optogenetic stimulation for 1.5 hours activated only neurons ipsilateral to the LED (top images), but not neurons contralateral to it (bottom images) (n = 3 mice). The white squares indicate the location of the enlarged areas on the right. (**C**). Delayed optogenetic stimulation started on day 15 post-tSCI (dotted line) resulted in significant increases in withdrawal threshold forces of bilateral paws at days 21 and 28 (n = 4–5 mice in each group, *p* < 0.05). However, corpus callosotomy on day 28 (black arrow) resulted in a loss of analgesic effect of the ipsilateral hind-paw, but not the contralateral hind-paw, to optogenetic stimulation (*p* < 0.05-0.001 when compared between ipsilateral and contralateral paws, repeated- measure ANOVA). Scale bars in A and B (left column): 500 µm; B (right column): 50 µm.

To further determine whether the bilateral analgesic effect of unilateral optogenetic stimulation was mediated by an excitatory drive carried by optogenetically stimulated layer V pyramidal neuron axons to the homotopic contralateral cortex through the corpus callosum, we tested the effect of transecting the corpus callosum between S1 regions of the two cortical hemispheres of tSCI + LED mice on day 28 post-tSCI. The transection of corpus callosum was confirmed by DiI stained axotomized axons in white matter in 3 mice. The result showed that the corpus callosotomy spanned ~4 mm rostroventrally and truncated interhemispheric axons between S1 of the two hemispheres (Fig. 6A, n = 3 mice). C-fos staining showed that optogenetic stimulation of unilateral S1 activated neurons of the ipsilateral cortex (Fig. 6B, left), but not the contralateral S1 (Fig. 6B, right), further confirming that the callosotomy eliminated the effect of optogenetic stimulation on activating neurons of the contralateral cortex.

To determine whether S1 optogenetic stimulation would be effective in reducing neuropathic pain that has already developed, we applied delayed optogenetic stimulation at day 14 post-tSCI. While optogenetic stimulation had no effect on the pain threshold in mice of the sham group, it caused significant increases in the withdrawal threshold of bilateral hind-paws in the tSCI group at days 21 and 28 post-injury (Fig. 6C. *p* < 0.05, repeated measure ANOVA followed by Bonferroni test, n = 4-5 mice), suggesting that S1 optogenetic stimulation has a treatment effect on neuropathic pain. Following the corpus callosotomy, the analgesic effect of the hind-paw that was contralateral to optogenetic stimulation remained unchanged, but the effect of the hind-paw that was ipsilateral to optogenetic stimulation disappeared (Fig. 6C. *p* < 0.01 and *p* < 0.001). This result indicates that enhancing excitatory activity *per se* is likely responsible for the observed trans-hemispheric analgesic effect.

### Optogenetic stimulation reduced neuronal intrinsic excitability and normalized synaptic transmission of layer V pyramidal neurons

**Figure 7:**
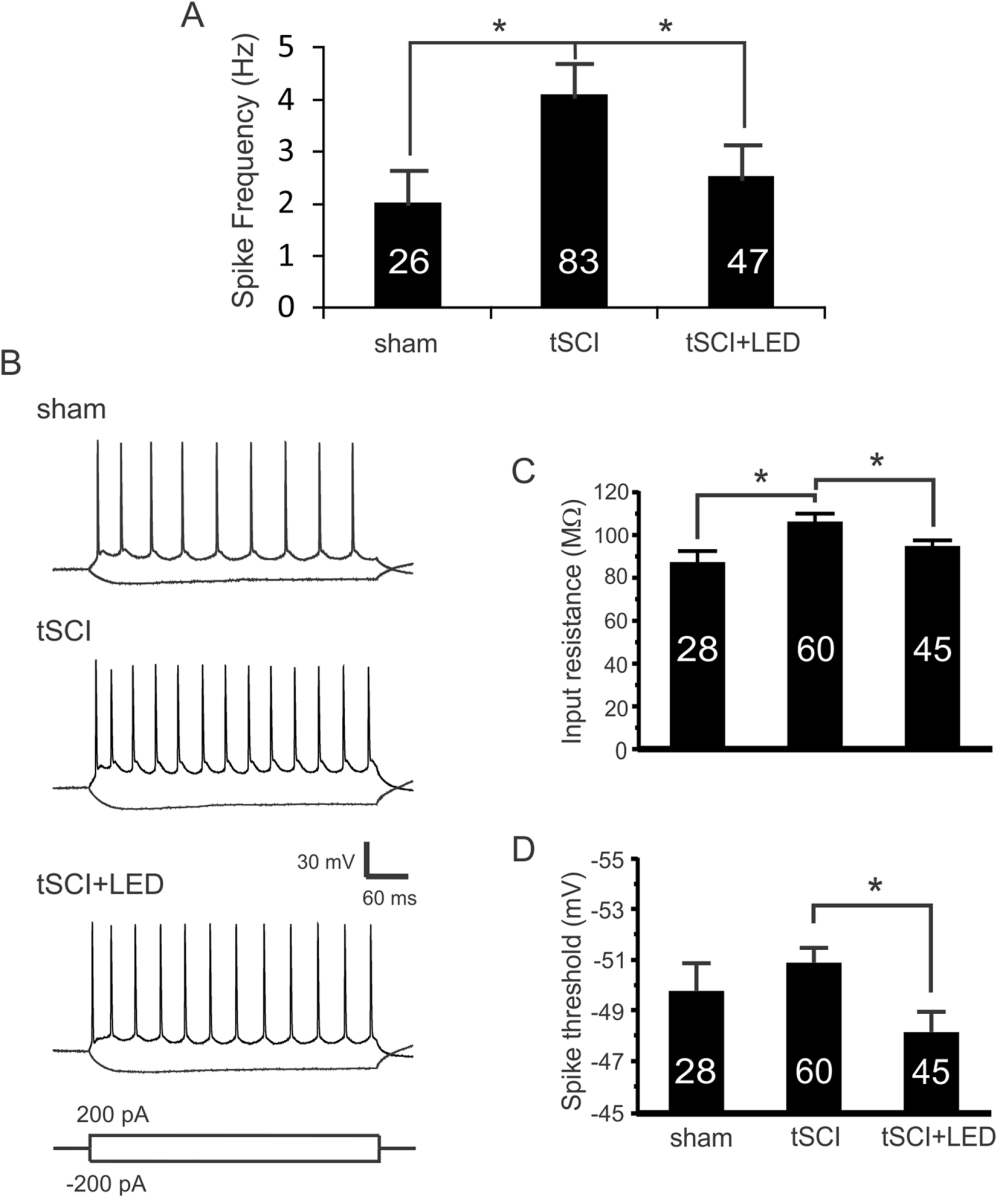
Optogenetic stimulation normalizes enhanced spontaneous firing and excitability of cortical layer V pyramidal neurons after tSCI. (**A**) Spike frequency of cortical layer V pyramidal neurons at 10 days after tSCI was significantly higher than that of the sham group, and was reduced after 5 days of optogenetic stimulation (sham: 2.0 ± 0.6 Hz; tSCI: 4.1 ± 0.6 Hz, tSCI + LED: 2.5 ± 0.6 Hz. *: *p* < 0.05, Mann-Whitney test). Numbers in the bars indicate neuron numbers in each group; same for the following figures. (**B**) Representative traces of AP firing of layer V pyramidal neurons in response to current injections. (**C**) Neuronal input resistance in tSCI group was higher than that of the sham group, but after optogenetic stimulation it was significantly lower than the tSCI group (sham: 86.6 ± 6.0 MΩ; tSCI: 105.5 ± 4.6 MΩ; tSCI + LED: 94.3 ± 3.3 MΩ. *p* < 0.05 when compared between sham and tSCI groups, and between the tSCI and tSCI + LED groups, One-way ANOVA followed by Bonferroni test). (**D**) Optogenetic stimulation resulted in a more positive active potential threshold than the tSCI group (−49.8 ± 1.1 mV, −50.9 ± 0.6 mV, −48.2 ± 0.8 mV in sham, tSCI, and tSCI + LED groups respectively. *p < 0.05 when compared between tSCI and tSCI + LED groups, One-way ANOVA followed by Bonferroni test).

To determine the mechanism of S1 optogenetic stimulation on cortical hyperexcitability, we recorded from layer V pyramidal neurons in tSCI mice after 5 days of cortical optogenetic stimulation. The spontaneous firing rate in tSCI group was significantly higher than the sham group, but it decreased significantly after optogenetic stimulation (Fig. 7A. 2.0 ± 0.6, 4.1 ± 0.6, and 2.5 ± 0.6 Hz for sham, tSCI, and tSCI + LED groups respectively, both *p* < 0.05, One-way ANOVA followed by Bonferroni test), suggesting reduced excitability of these neurons after optogenetic stimulation.

We analyzed neuronal membrane properties under current clamp mode (Fig. 7B). There was no significant difference in resting membrane potentials among all groups (−63.6 ± 0.4, −63.1 ± 0.3, −63.9 ± 0.4 mV for the sham, tSCI, and tSCI + LED groups respectively, *p* > 0.05, One-way ANOVA), but there was a significant increase in input resistance after tSCI, which was decreased after optogenetic stimulation (Fig. 7C. 86.6 ± 6.0, 105.5 ± 4.6, and 94.3 ± 3.3 MΩ for sham, tSCI, and tSCI + LED groups respectively, both *p* < 0.05). Furthermore, the tSCI + LED group had a more positive AP threshold than the tSCI group (Fig. 7D. −49.8 ± 1.1, −50.9 ± 0.6, and −48.2 ± 0.8 mV for sham, tSCI, and tSCI + LED groups respectively, *p* < 0.05, One-way ANOVA followed by Bonferroni test). These results indicate that these changes in intrinsic properties induced by optogenetic stimulation contribute to reversing hyperexcitability of cortical pyramidal neurons after tSCI.

**Figure 8:**
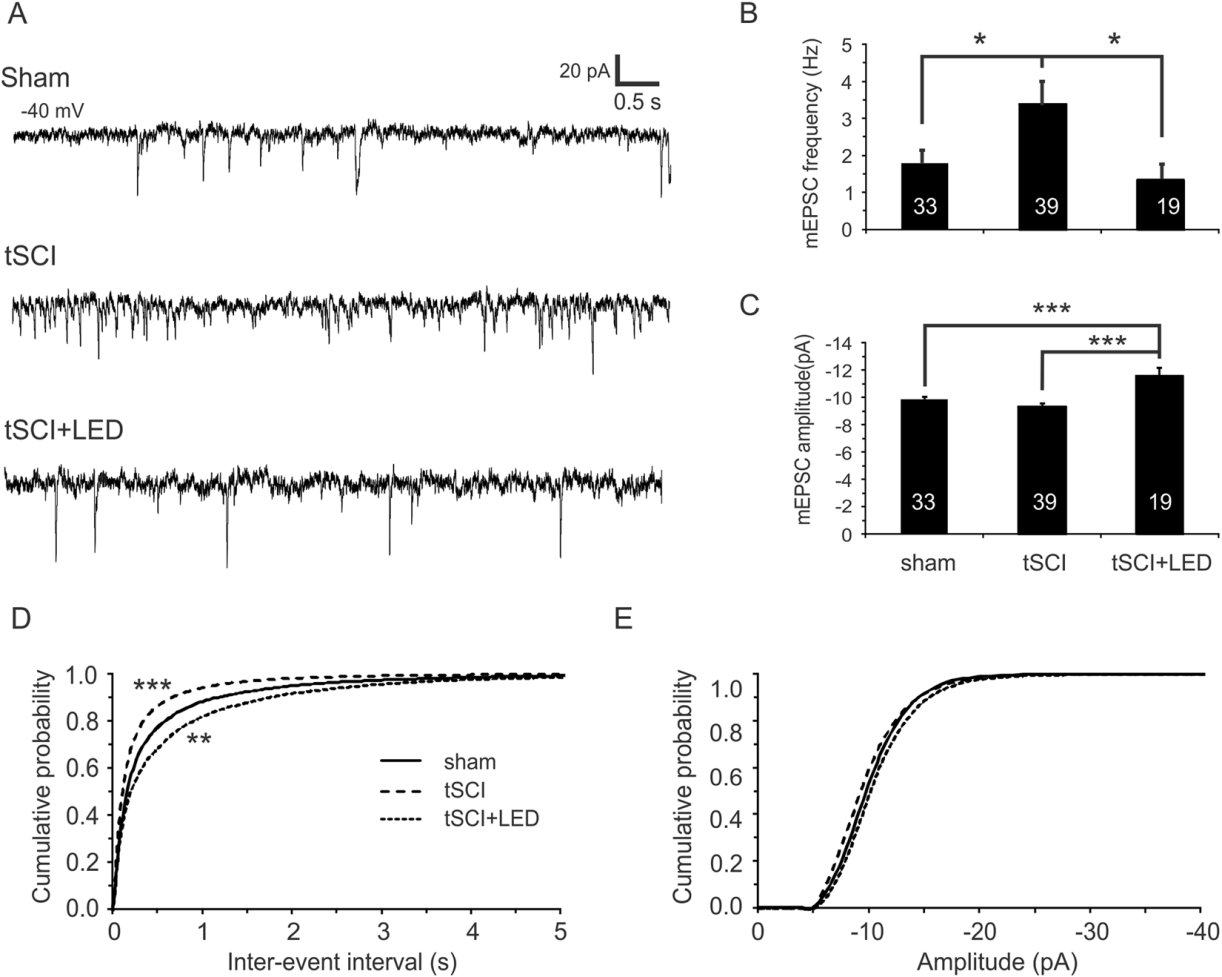
Cortical optogenetic stimulation normalizes the elevated mEPSC frequency but increases mEPSC amplitude of layer V pyramidal neurons in tSCI mice. (**A**) Representative mEPSC traces from cortical layer V pyramidal neurons of the sham, tSCI, and tSCI + LED groups. (**B**) mEPSC frequency in tSCI group was significantly higher than the sham group, but became normal after optogenetic stimulation for 5 days (sham: 1.8 ± 0.4 Hz; tSCI: 3.4 ± 0.5 Hz; tSCI + LED: 1.3 ± 0.4 Hz. *p < 0.05, one-way ANOVA followed by Tukey test). (**C**) tSCI resulted in no significant change in the amplitude of mEPSC (*p* > 0.05), but optogenetic stimulation caused a significant increase in the amplitude of mEPSC (Sham: −9.8 ± 0.2 pA; tSCI: −9.4 ± 0.2 pA; tSCI + LED: −11.6 ± 0.6 pA. ***p < 0.001 when tSCI + LED group was compared with the sham or tSCI groups). (**D**) Cumulative histograms of inter-event intervals. The leftward shift of the tSCI curve indicates reduced inter-event intervals, and the rightward shifts of the tSCI + LED indicates increased inter-event intervals after stimulation (***p < 0.001 and **p < 0.01 for tSCI and tSCI + LED groups when compared to the sham group respectively, K-S test). (**E**) There were no significant differences in cumulative distribution of mEPSC amplitude among the 3 groups (*p* > 0.05).

Homeostatic plasticity often involves changes in excitatory and inhibitory synaptic transmission^10,26–28^. We recorded miniature excitatory and inhibitory postsynaptic currents (mEPSCs and mIPSCs) from layer V pyramidal neurons of the S1. The mEPSC frequency of the tSCI group was significantly higher than that of the sham group, but it was reversed after optogenetic stimulation (Fig. 8A,B. 1.8 ± 0.4, 3.4 ± 0.5, and 1.3 ± 0.4 Hz for sham, tSCI, and tSCI + LED groups respectively, *p* < 0.05 when sham and tSCI or tSCI and tSCI + LED were compared). Interestingly, the amplitudes of mEPSCs were similar in the sham and tSCI groups, but it became larger after optogenetic stimulation (Fig. 8C. −9.8 ± 0.2, −9.4 ± 0.2, and −11.6 ± 0.6 pA for sham, tSCI, and tSCI + LED groups respectively, *p* < 0.001, one-way ANOVA followed by Bonferroni test). The cumulative probability curve of inter-event intervals had a left shift for the tSCI group relative to the sham group (Fig. 8D. *p* < 0.001, Kolmogorov–Smirnov [K-S] test), but a right shift for the tSCI + LED group (*p* < 0.005). There were no changes in mEPSC amplitude curves among all 3 groups (Fig. 8E, *p* > 0.05). Analyses of the rise time and decay time constant of mEPSC in the 3 groups showed no significant differences among them (data not shown).

**Figure 9:**
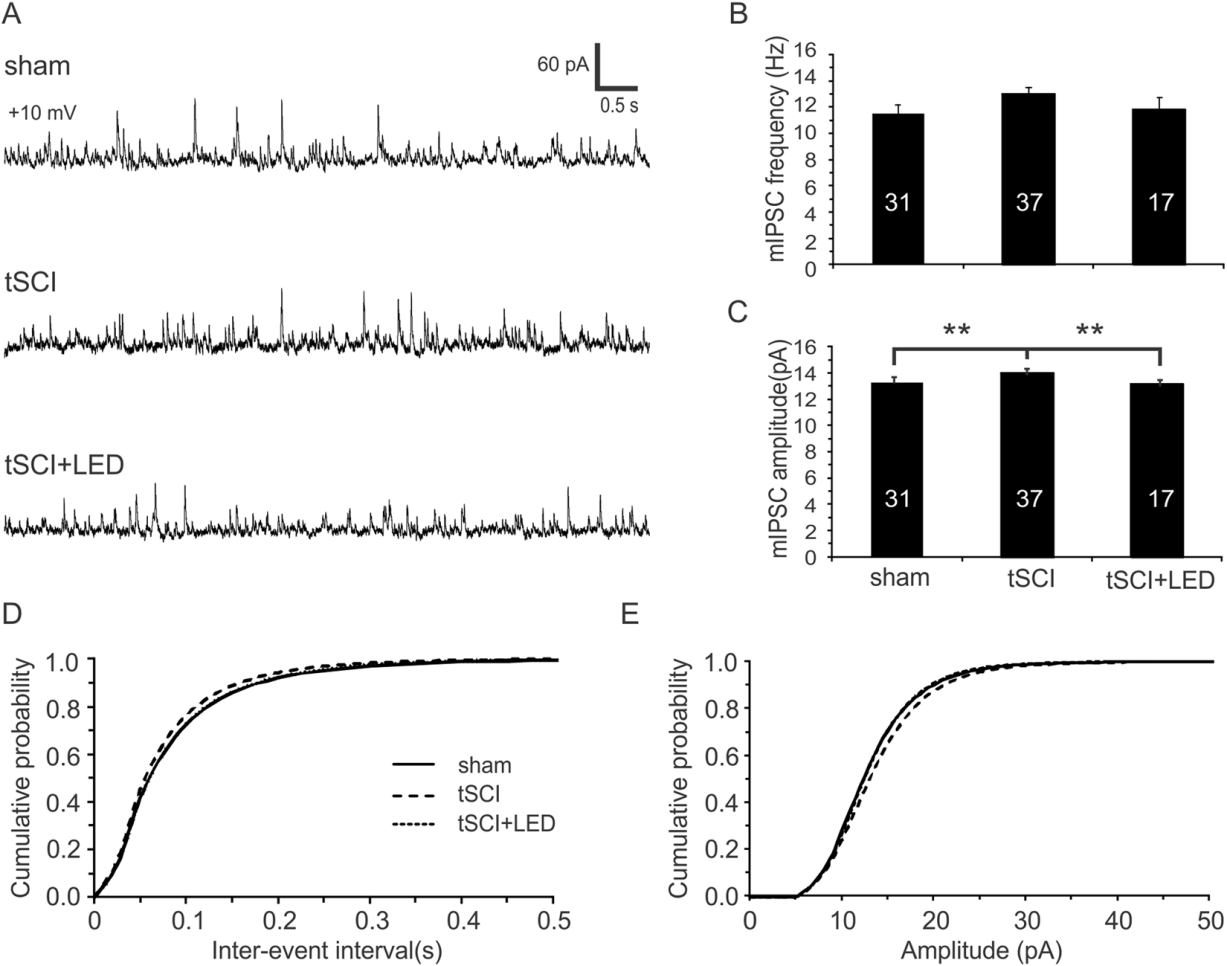
Cortical optogenetic stimulation normalizes the increased mIPSC amplitude of layer V pyramidal neurons in tSCI mice. (**A**) Representative mIPSC traces of the 3 groups at a holding potential of + 10 mV. (**B**) There were no significant differences in the mean frequency of mIPSCs among all groups (sham: 11.5 ± 0.7 Hz; tSCI: 13.0 ± 0.5 Hz; tSCI + LED: 11.8 ± 0.9 Hz. *p* > 0.05). (**C**). The amplitude of mIPSC in tSCI group was significantly higher than the sham group (Sham: 13.3 ± 0.2 pA; tSCI: 14.0 ± 0.2 pA. *p* < 0.01), which became decreased in the tSCI + LED group (tSCI + LED: −13.1 ± 0.2 pA. *p* < 0.01 when the tSCI and tSCI + LED groups were compared). (**D-E**). There were no significant differences in cumulative distributions of mIPSC frequencies and amplitudes (*p* > 0.05, K-S test).

We recorded mIPSCs at a calculated mEPSC reversal potential of +10 mV (Fig. 9A). There were no significant differences in mIPSC frequencies (Fig. 9B. *p* > 0.05, one-way ANOVA), but mIPSC amplitude of the tSCI groups was significantly higher than that of the sham group, which became normal after optogenetic stimulation (Fig. 9C. both *p* < 0.01, one-way ANOVA followed by Bonferroni test). There were no differences in cumulative probability curves of both mIPSC inter-event interval and amplitude among all groups (Fig. 9D,E. *p* > 0.05).

### Pharmacological enhancement of excitatory activity also reduced neuropathic pain

**Figure 10:**
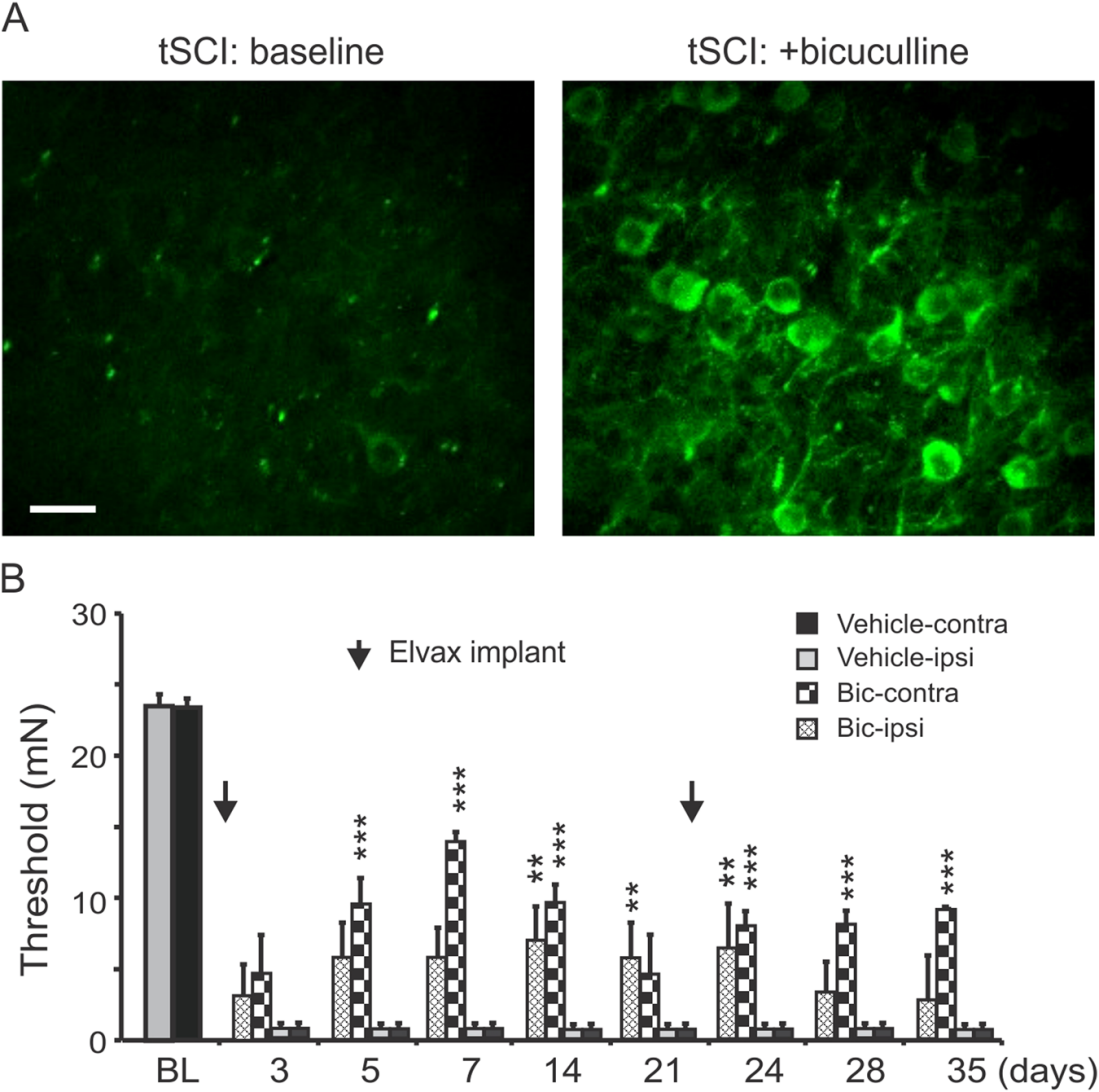
Pharmacological enhancement of cortical excitatory activity also reduces mechanical hypersensitivity. (**A**). *In vivo two* photon images from a GCaMP6 tSCI mouse show low spontaneous activity at baseline (left) and dramatically enhanced activity after applying 20 mM bicuculline solution on the S1 cortical surface. The bicuculline application induced highly synchronized rhythmic epileptiform activities of the cortex, suggesting that GABA_A_ receptor activation is inhibitory (n = 2 mice). Scale bar: 20 µm. (**B**). Implanting bicuculline-Elvax (200 µm thick) on the pial surface of the S1 at day 1 and day 21 post-tSCI (black arrows) significantly increased the withdrawal threshold of bilateral paws (n = 4 in blank-Elvax and n = 6 in bicuculline-Elvax group). **p < 0.01; ***p < 0.001.

The homeostatic plasticity hypothesis suggests that chronic pharmacological enhancement of cortical activity may also reduce neuropathic pain. To test this hypothesis, we used a GABA_A_ receptor antagonist bicuculline to enhance cortical activity. Because neuronal injury can cause a positive shift of GABA_A_ reversal potential and excitatory GABAergic response, we first used *in vivo* two-photon imaging in GCaMP6 mice to directly determine whether bicuculline would increase cortical activity. Application of 20 mM bicuculline solution on the pial surface caused a dramatic increase in cortical spontaneous activity, which soon turned into highly synchronized, rhythmic epileptiform activities (Fig. 10A, n = 2 mice). The result indicates that bicuculline enhances cortical activity in the tSCI model.

We implanted on the S1 pial surface a slice of drug-releasing polymer Elvax that contained bicuculline or vehicle (saline) on the second day following tSCI. In contrast to mice that received saline-containing Elvax implants in which the implants had no effect on the pain threshold, mice that received bicuculline-containing Elvax implants had significantly higher pain thresholds on bilateral hind-paws (Fig. 10B). The analgesic effect of the bicuculline-Elvax group peaked at day 7 post-tSCI, and then gradually decreased in 2 weeks, likely due to drug depletion over time. A second implant of bicuculline-Elvax at day 21 post-tSCI maintained the analgesic effect for >2 additional weeks (Fig. 10B, *p* < 0.01-*p* < 0.001, Repeated measure ANOVA followed by Bonferroni test, n = 4 in blank-Elvax and n = 6 in bicuculline-Elvax group). Similar to optogenetic stimulation, unilateral bicuculline-Elvax implant has a bilateral analgesic effect. The result suggests that enhancing cortical activity by blocking GABA_A_ receptor is effective in reducing mechanical hypersensitivity.

## Discussion

Our data demonstrated homeostatic regulation of neuronal activity in the S1 following tSCI *in vivo* and *in vitro*, and the contributions of increased intrinsic excitability and mEPSC frequency to hyperexcitability of layer V pyramidal neurons. We found that optogenetic or pharmacological enhancement of cortical excitatory activity is sufficient to reduce the mechanical hypersensitivity associated with neuropathic pain. We further showed that optogenetic stimulation is able to effectively normalize neuronal intrinsic properties and synaptic transmission in the tSCI model. To our knowledge, this is the first demonstration that homeostatic activity regulation in the S1 plays a role in the mechanism of neuropathic pain and that optogenetic or pharmacological enhancement of S1 activity is effective in diminishing behavioral changes commonly observed with neuropathic pain.

We targeted the S1 because of its critical involvement in pain perception^29^. Increasing evidence suggests that S1 is involved in nociceptive perception in both rodents^30,31^ and human^32^. Specifically, S1 plays a prominent and highly modulated role in nociception, including coding location, intensity, and duration of pain sensation^29,33^. S1 synaptic plasticity has been demonstrated in models of neuropathic pain and S1 activation is associated with pain perception^12,31,34–37^, while inhibiting S1 plasticity or activity has the effect of reducing or preventing the development of pain^36,37^.

Retina lesion or monocular visual deprivation has been shown to cause homeostatic activity regulation in the adult visual cortex^10,38^. Transient spinal cord ischemia is known to cause motor neuron death, white matter lesion, and inflammatory responses of the affected spinal cord segments^18,39–41^. Impaired motor function lasts for a few days followed by recovery^42^. Together with the long-lasting pain-like behavior, these pathological changes suggest likely involvements of damage to the nociceptive pathways in this model. Our finding of initial activity loss after tSCI followed by a state of hyperexcitability in S1 supports homeostatic activity regulation. Similarly, traumatic spinal cord injury also results in slower and more silent cortical spontaneous activity in the deafferented cortex as well as in the neighboring cortex during an earlier time period^11,13^. Weeks later, neurons in layer IV and V of the S1 have higher spontaneous firing rates and an increased tendency to fire bursts of APs^43^, and electrical stimulation induces a stronger and enlarged area activation of the S1^12^. These sequential changes in neuronal activity support homeostatic regulation of activity in the S1 following tSCI.

It appears that changes in intrinsic properties and synaptic input both contribute to S1 hyperexcitability. The increased input resistance of the neurons will make their received synaptic input more efficient in changing membrane potential. A ~90% increase in the frequency and unchanged amplitude of mEPSCs will greatly enhance excitatory drive to the pyramidal neurons likely through a presynaptic mechanism, while the increase (~5%) in mIPSC amplitude was small. Together, the net effect of these changes is a shift towards enhanced excitation of the cortical network in the cortex following tSCI.

Sensory deprivation also causes plasticity and reorganization of the S1, which may be related to the development of neuropathic pain^37,44,45^; reversing it is effective in relieving neuropathic pain behavior^46^. However, such reorganization is not contradictory with the homeostatic mechanism we propose here, because neuronal activity and plasticity intricately connect to each other and changes in activity often trigger cortical reorganization. For example, visual deprivation causes changes not only in neuronal activity, but also in dendrites and spines^10^. Cortical deafferentation causes synchronized slow wave activity, which promotes axon sprouting^47^.

Direct electrical stimulation of the S1 has been shown to cause pain sensation in human subjects during surgery^48^, which is apparently contradictory to our results that optogenetic stimulation of the S1 reduced pain-like behavior. However, there are several major differences between optogenetic stimulation and the effect of S1 electrical stimulation. First, the neuronal populations being activated are different. Optogenetic stimulation in our experiment activated only cortical excitatory neurons, with the majority of these being layer V pyramidal neurons, which is in contrast to activation of both pyramidal and interneurons by intracortical electrical stimulation. Second, the number of neurons being activated by the optogenetic stimulation is probably larger and more diffusely distributed than that evoked by electrode stimulation. Third, more importantly, the chronic effect of S1 optogenetic stimulation occurred after hours of cortical stimulation, and the effect peaked in 3–5 days, which is in contrast with intraoperative acute S1 stimulation that exacerbated pain in human patients. Furthermore, our electrophysiological data showed that chronic optogenetic stimulation of the S1 actually reduced S1 excitability through decreasing spontaneous AP firing, increasing AP threshold, and greatly reducing excitatory synaptic input (Figs 5–6). The effect of corpus callosotomy on eliminating optogenetically induced analgesia (Fig. 6) further supports a role of activity enhancement on relieving pain. Interestingly, a recent study showed that optogenetic stimulation of S1 activity using beta and gamma frequencies acutely block pain transmission^49^. While motor cortical stimulation is a common treatment for refractory chronic pain including neuropathic pain and phantom pain, S1 stimulation is also shown to be effective in relieving pain in human and animal studies^50,51^.

Our findings suggest that chronic optogenetic stimulation normalizes electrophysiological alterations that underlie cortical hyperexcitability in the tSCI model. Because the layer V pyramidal neurons have a dominant effect in determining activity level of cortical columns and our optogenetic stimulation on the ChR2 transgenic mice mainly activates these neurons, our patch clamp recordings were made from these neurons. Although layer V pyramidal neurons and layer II/III neurons (which we imaged) have different layer-specific functions in neural coding, they are interconnected to function as cortical columns and their activity is often synchronized with similar firing frequency^52,53^. Therefore, changes in activity and excitability detected in layer II/III and layer V are likely consistent. The reduced spontaneous firing could be attributed to increased AP threshold, decreased input resistance, and reduced mEPSC frequency. Although the optogenetic stimulation also increased the amplitude of mEPSC, such increase was small in contrast to the great decrease in mEPSC frequency (Fig. 6B,C). Similarly, the decrease in the amplitude of mIPSCs after optogenetic stimulation was also small and the frequency was unchanged (Fig. 7B,C). Thus, the net effect of optogenetic stimulation on pyramidal neurons is a significant reduction in excitatory synaptic drive, which would decrease network excitability and cortical activity. Consistently, electrical stimulation or bicuculline treatment of cortical neurons also decreases spontaneous and burst spike activity and reduces evoked EPSP strength^54,55^. Therefore, enhancing cortical activity is correlated with homeostatic reduction of spontaneous firing and neuronal excitability.

The effectiveness of chronic cortical release of bicuculline in relieving neuropathic pain (Fig. 8) is in contrast to a generally accepted concept that inhibiting cortical excitation relieves neuropathic pain. For example, cortical infusion of GABA_A_ receptor agonists (e.g. muscimol or GABA) relieves pain, while acute cortical injection of GABA_A_ receptor antagonists induces burst spiking and pain^35,56,57^. These apparently conflicting findings suggests that two opposite strategies may be used for controlling neuropathic pain. One strategy is to directly suppress cortical hyperexcitability in neuropathic pain by directly suppressing network excitatory activity or enhancing inhibition, such as the analgesic effect of locally applied muscimol or the use of specific antiepileptic drugs for pain treatment^4,58^. The other strategy we propose here is to enhance cortical excitatory activity so that the homeostatic mechanism takes effect and intrinsically reduces neuronal hyperexcitability. The effectiveness of cortical stimulations on neuropathic pain may be explained by this mechanism^17^. Similarly, a recent study showed that pharmacologically or optogenetically stimulating dopamine neurons of the ventral tegmentum can homeostatically reduce neuronal hyperactivity and reverse depression related behavior^59^.

Although we show that enhancing cortical activity is sufficient to reduce network hyperexcitability and control neuropathic pain, involvements of other mechanisms such as activations of brain inhibitory pathways and descending projections cannot be ruled out^16,60^. Such changes may result from descending activation of subcortical nuclei and dorsal horn of the spinal cord and/or from reactions of pain pathways to reduced cortical excitability following activity enhancement. Given the complexity and interconnectivity of the nociceptive system in the brain and spinal cord, dissecting out these potential pathways is beyond the scope of this study.

Controlling nociceptive hyperexcitability is a major strategy for controlling neuropathic pain. However, directly suppressing such hyperexcitability has unsatisfactory efficacy^57^. By demonstrating a role of homeostatic plasticity in neuropathic pain, we provide the first evidence that enhancing cortical excitatory activity *per se* is sufficient to reduce network hyperexcitability and relieve neuropathic pain, which may not only provide a mechanistic explanation for the effectiveness of cortical stimulation in controlling refractory neuropathic pain, but also reveal a novel strategy for developing pharmacological treatment for neuropathic pain. Furthermore, enhancing cortical activity may be beneficial in controlling other neurological disorders featuring deafferentation-initiated cortical hyperexcitability, such as phantom limb pain, tinnitus, and acquired epilepsy^9,27^. Indeed, repetitive transcranial magnetic stimulation and electrical cortical stimulation are found promising for controlling these conditions^51,61,62^.

## Materials and Methods

### Animal models of neuropathic pain and Von Frey test

All experimental procedures were approved by the Animal Care and Use Committee of the Institutional Guide for the Care and Use of Laboratory Animals at Indiana University School of Medicine. Animal procedures adhered to guidelines outlined within the Guide for and Care and Use of Laboratory Animals of the National Institutes of Health, with ethical guidelines outlined by the International Association for the Study of Pain. All behavioral data collection and analysis was performed by blinded individuals. Tibial nerve injury (TNI) and tSCI models were performed on adult (6–8 weeks old) male Thy1-Channelrhdopsin 2 (ChR2) transgenic mice.

The mice were anesthetized with ketamine/xylazine (87.7/12.3 mg/kg, i.p.). For TNI, after a small incision was made on the left leg to expose the tibial nerve, a silk thread was used to make three consecutive loose ligations around it and the nerve was transected distal to the ligation. For tSCI, the abdominal cavity of the mice was opened and the abdominal aortic artery was exposed at the level of the left renal artery. The aortic artery was then isolated and blocked with a metal clip (vascular micro bulldog) for 60 minutes. Mice in the sham groups received similar surgical procedures without actual nerve ligation (in TNI) or aortic occlusion (in tSCI).

Mechanical hypersensitivity was measured using a mechanical Von Frey anesthesiometer. Calibrated Von Frey filaments measuring 100 μm in diameter capable of exerting bending forces of 5, 10, 20, 40, 60, 80, and 120 mN were applied to the plantar surface of the animal hindpaw^63^. Each stimulus lasted approximately 1 second and had a 10–15 second intermission between applications. Increasing Von Frey filaments were applied until a paw withdrawal was observed and the corresponding stimulation intensity was recorded. The test was conducted by a research analyst blinded to treatments and group assignment.

### *In vivo* two-photon imaging for evaluating neuronal activity, effects of bicuculline, and optogenetic stimulation on cerebral circulation

Under ketamine/xylazine (87.7/12.3 mg/kg, i.p.) anesthetization, cranial windows were prepared in adult GCaMP6s transgenic mice and were centered at −2.5 mm bregma and 2.5 mm lateral, using a previously described technique^64^. For imaging, the mice were sedated with chlorprothixene^35^, and calcium transients of cortical layer II/III neurons were imaged at 4-5 frames per second for 3 minutes using a two-photon microscope (Prairie Technologies)^10^. Excitation was provided by a tunable Maitai Ti:sapphire laser (Newport, Mountain View, CA) tuned to 900 nm. Band-pass filtered fluorescence (560–660 nm) was collected by photomultiplier tubes of the system. The same fields were identified and imaged at all different time points.

For data analysis, neurons were manually selected based on average projections of image stacks combined with looking through the whole image stacks. Raw fluorescence (*F*) was measured as average fluorescence of pixels lying within a cell in each frame and background fluorescence (*F*_*o*_) was measured as mean of 2 sites that were just around the cell and contained no neuronal structures. ΔF/F signals were calculated as (*F*- *F*_*o*_ *)/F*_*o*_. Cell activity was measured by both mean integrated fluorescence and ratio of active neurons^10^. Integrated fluorescence was calculated by integrating all fluorescence above a threshold of 20% ΔF/F. The threshold was used to eliminate possible influence of noise. Active neurons were defined as cells that had fluorescence intensity above threshold level at least once. The ratio of active neurons at a given time period was calculated by dividing the number of active cells by the total number of cells in that particular imaging field^10^. Both values of each mouse at different time points were normalized to the baseline levels.

To determine whether bicuculline had an excitatory or inhibitory effect on cortical pyramidal neurons of neuropathic pain model, we prepared tSCI in three GCaMP6 mice as described above. On day 3 after the surgery, the mice were anesthetized with ketamine/Xylyzine and acute cranial windows were made over the S1 area. Following baseline imaging of activity of layer II/III GCaMP neurons, 20 mM bicuculline was applied onto the pial surface, and the same imaging fields were repeatedly imaged every 15–30 minutes for up to 5 hours.

To evaluate the effect of light stimulation with blue LED on cerebral blood circulation, four C57BL mice were used for *in vivo* two-photon imaging. Cranial windows were prepared using the same technique as described above. Two weeks later, the mice were sedated by an intraperitoneal injection of chlorprothixene, and followed by an intraperitoneal injection of 200 µL 0.4 mg/mL Sulforhodomine 101 at 15 minutes before imaging to label the blood plasma. Arterioles and venules (20–30 µm diameters) were identified based on vessel shape, direction of blood flow relative to branching pattern, and flow velocity. For each mouse, two arterioles and two venules on the S1 were imaged in both frame (512 × 512 pixels) and line scan (1 millisecond [ms] per line) modes using PrairieView software of the two-photon system. Following a baseline imaging, the same light pulses used for optogenetic stimulation were applied to the cranial window for 15 minutes. Then the same blood vessels were imaged again to compare potential changes in vessel diameter and red blood cell (RBC) velocity after light stimulation. Imaging analyses were made with Metlab and NIH ImageJ software. Vessel diameters were manually measured from the maximum distance of longitudinal sections of the vessels. RBC velocity was determined from line-scan images using a Metlab script based on a technique described by Drew *et.al*.^65^.

### Cortical optogenetic stimulation, virus injection, and corpus callosotomy

For optogenetic stimulation, the skull above the S1 was exposed and the periosteum was cleaned. After a layer of cyanoacrylate glue was applied, a circular area of the skull on top of the S1 region (~3 mm diameter) was removed and a circular cover glass was sealed onto the edge of the skull to make a cranial window. A high-intensity blue LED was then glued on top of the cover glass, with a circular area of ~2.5 mm diameter being exposed to the light and the outer region being blocked with a piece of opaque tape. The LED was controlled by a miniature circuit that generated a burst of 3 pulses at 33 Hz once every 3 seconds. Two sessions of 3-hour stimulation with a 2-hour break in between were applied daily to the contralateral S1 on the second day following TNI or tSCI for 1–3 weeks.

For focal infection of ChR2 virus, 1 µL of an AAV1.CaMKIIa.hChR2(H134R)-mCherry.WPRE.hGH (Addgene26975, purchased from the Penn Vector Core of the University of Pennsylvania School of Medicine) was injected into the center of HL-S1 area using a glass pipette. After the pipette was inserted to about 650 µm below the pial, injection was made with a microinjection system in 10 minutes. The pipette was then kept in position for another 10–15 minutes. Optogenetic stimulation was applied in 2 weeks after viral injection, using the same protocol with two sessions of 3 hours for 7 days.

Corpus callosotomy was made by modifying a previously described technique^66^. After anesthetization, a 4 mm long slit was drilled in the skull, at 0.5 mm lateral to the midline and parallel to the S1. An “L”-shaped bent 34-gauge needle was inserted into the midline between two cortical hemispheres and lowered vertically 1.5 mm into cortical tissue to cut the corpus callosum, then lifted to underneath the pia and withdrawn. To visualize the cutting path and transected axons from callosotomy, a needle that was coated with a thin layer of lipophilic fluorescent dye DiI was used to make the callosotomy in 3 mice. The mice were sacrificed, perfused, and fixed in 48 hours after the surgery. Coronal cortical slices (50 µm) were cut and imaged under a fluorescence microscope.

### C-fos staining

Four mice were assigned to each of the naïve, naïve + LED, and tSCI + LED groups. For the LED groups, the mice received 1.5 hours of optogenetic stimulation using the protocol described above. Then, the mice were immediately anesthetized with sodium pentobarbital (150 mg/mL) and transcardially perfused with 50 mL of 0.9% saline, followed by 50 mL of 4% paraformaldehyde. After the brains were removed and fixed overnight at 4 °C, they were transferred to a 30% sucrose solution and stored at 4 °C until isotonic with the solution. A freezing microtome (Leica Biosystems, IL) was used to cut coronal sections of the S1 to 30 μm thickness. The sections were placed in cryoprotectant (30% glycerol and 30% ethylene glycol of 0.1 M phosphate buffer). After the sections were washed 3 times in 0.1 M phosphate buffer solution (PBS), they were incubated overnight at room temperature in 1:700 rabbit polyclonal anti-c-fos antibody (Catalog No. ABE457, Millipore, MA), followed by incubation for 2 hours at room temperature in 1:200 donkey anti-rabbit antibody. The sections were imaged under a florescence microscope.

### Preparation and implantation of drug-containing Elvax

Elvax implants were prepared as described previously^67–69^. Briefly, ethylene vinyl acetate copolymer (EVA) (40% vinyl acetate by weight, DuPont, St. Louis, USA) was washed in 100% ethanol several times and dissolved in dichloromethane to give a 10% solution (w/v). A 100 µL sample of double-distilled water (for blank implants) or an aqueous solution of bicuculline methiodide was added to the Elvax/dichloromethane solution. The final concentration of bicuculline was 10 mM. The suspension was vortexed for 30 minutes and poured into a glass tube and placed on dry ice for 30 minutes. After storage at −20 °C for 7 days, the Elvax was cut into 200 µm slices using a vibratome and stored at −20 °C until use.

For implantation, mice were anesthetized with ketamine/xylazine (87.7/12.3 mg/kg, i.p.). After a craniotomy was made over the left S1 region, a slice of Elvax (2 × 2 mm) containing vehicle or drug was placed on the cortical surface to cover the S1 area. The craniotomy was covered with a piece of sterile plastic wrap, and the skin was sutured.

### Recording cortical sensory evoked potentials *in vivo* and patch clamp recording *in vitro*

Cortical sensory-evoked potentials were recorded from the HL-S1 *in vivo*. In anesthetized C57BL mice, small holes (~0.5 mm) were drilled on the skull above HL-S1 (1 mm posterior to bregma and 1.8–2 mm lateral to the midline) and above the cerebellum. Stainless steel screws were implanted into these holes as recording and reference electrodes respectively. Baseline responses were recorded at 7–10 days after screw installation in awake animals. To induce sensory-evoked potentials, an acupuncture needle was inserted into the paw of the contralateral hindlimb. Electric stimulus (0.2 ms square pulses at 0.5 mA, once every 20 seconds) was applied and gradually increased until a response was induced. Twice the level of this threshold current was used for inducing cortical evoked potentials. The responses were measured from an average of 20 traces. Following the baseline recordings, the mice received tSCI surgery, followed by optogenetic stimulation on the second day after the surgery for 5 days.

For *in vitro* patch clamp recording, brain slices (350 µm thick) containing HL-S1 were cut and maintained using previously described procedures^70^. Patch clamp recordings were made at room temperature (22-23 °C) from layer V pyramidal neurons. Glass patch electrodes had an impedance of 3–5 MΩ when filled with intracellular solution containing (in mM): 20 KCl, 100 cesium gluconate, 10 HEPES, 4 Mg-ATP, 0.3 Na-GTP, 10 sodium phosphocreatine and 3 QX-314. For current clamp recordings, K-gluconate based solution was used, which contained (in mM): 100 K-gluconate, 20 KCl, 10 HEPES, 4 Mg-ATP, 0.3 NaGTP and 10 sodium phosphocreatine. To measure spontaneous action potential (AP) firing rate, current clamp recordings were made by adjusting the membrane potentials to −60 mV or −65 mV and in a modified artificial cerebrospinal fluid (ACSF) that contained (in mM): 124 NaCl, 3.5 KCl, 0.5 MgCl2, l.25 NaH2PO4, 26 NaHCO3, 1 CaCl2, and 25 Dextrose^23^. For measuring miniature excitatory and inhibitory postsynaptic currents (mEPSCs and mIPSCs), 1 µM tetrodotoxin (TTX) was added into the ACSF. The reversal potentials were determined by voltage clamping the postsynaptic neurons to different potentials (in 5 mV increments) between −55 and −30 mV for mIPSCs and between −5 and +15 mV for EPSCs. With the above internal solutions and modified ACSF, the measured average reversal potentials were 11.3 ± 1.2 mV for mEPSCs and −40.5 ± 1.8 mV for mIPSCs respectively.

Intrinsic properties and AP firings were determined from neuronal responses to a series of 500 ms hyperpolarizing and depolarizing currents pulses (25–50 pA steps) under current clamp mode. Analysis of intrinsic properties was done using Clampfit 9.0 software. Resting membrane potential was measured as the membrane voltage after break-in and no current was injected. Liquid junction potential was not adjusted. Input resistance was determined from the slope of a best-fit-line through the linear segment of a voltage-current relationship. The first AP elicited by the lowest current injection was used for measuring AP properties. AP threshold was determined at the voltage level when voltage deflection exceeded 10 mV/ms. Spontaneous AP spikes, mEPSCs, and mIPSPs were detected using event detecting software based on the rise time, event width, minimal amplitude, and event duration^71^. The detector parameters were manually adjusted and tested to ensure a >95% detection of all events. Cumulative probability distributions were plotted by randomly selecting 100 events from each neuron in each group.

### Statistics

Data are presented as mean±SEM. Statistical significances were determined using nonparametric Mann-Whitney test for spike rate, two-way repeated measure analysis of variance (ANOVA) for *in vivo* calcium imaging data and pain threshold, and one-way ANOVA for mean amplitude and frequency of mEPSCs and mIPSCs, followed by appropriate *post hoc* tests. Statistical significance was set as *p* < 0.05. Comparisons of cumulative distributions of synaptic events were made with Kolmogorov-Smirnov test (K-S test).
